# Attention to detail: A photo‐elicitation study of salience and packaging design for portion control and healthy eating

**DOI:** 10.1111/nbu.12588

**Published:** 2022-11-03

**Authors:** Ruiqi Chu, Tang Tang, Marion M. Hetherington

**Affiliations:** ^1^ School of Design University of Leeds Leeds UK; ^2^ School of Psychology University of Leeds Leeds UK

**Keywords:** design, downsizing, food packaging, photo‐elicitation, portion control, salience

## Abstract

Evidence demonstrates that food packaging attracts consumers to purchase and has the potential to nudge consumers towards healthy choices, including reducing portion size. However, food purchasing decisions are often automatic and packaging features may go unnoticed. Therefore, it is important to understand what consumers identify as most salient about packaging: what they notice and why, and which elements might nudge consumers towards healthy options and smaller portions of high‐energy‐density foods. This study explored consumer perception of food packaging, investigated specific features associated with portion control and elicited design ideas to improve packaging for healthy eating and downsizing. A qualitative approach was adopted applying a participant‐driven photo‐elicitation (PDPE) task with in‐depth interviews. Participants were 25 adults living in the UK (aged 20–32 years; 17 females, 8 males, $\bar{x}$BMI = 23 kg/m^2^). Participants took photographs of 10 food packages according to salience (*n* = 5) and portion control (*n* = 5). These were uploaded to a secure site and then discussed at the interview, which was transcribed and analysed. The salience of packaging was described in terms of trust building, stimulating appetite and relating to self‐identity, whereas for portion control, themes included structural reminders, healthy prompts and portion awareness. Packaging can be designed to make health value or serving size more salient by prompting portion control and increasing the attractiveness of packaging. While food purchase decisions happen with little deliberation, when probed, consumers provide useful insights for packaging design to assist portion control.

## BACKGROUND

Increasing prevalence of obesity and other nutrition‐related non‐communicable diseases worldwide (Chooi et al., 2019; Doak, 2002; Stanhope, 2016) is partly attributed to excess intake of high‐energy‐density (HED), nutrient‐poor foods (Drewnowski & Popkin, 1997; Vecchio & Cavallo, 2019; Yanovski, 2018). Consuming a healthy diet is desirable, but widespread availability of HED foods, which are appealing and affordable, compete with achieving a healthy diet. Adherence to dietary goals is challenging in the current environment (Marteau et al., 2012). However, food environments can be shaped to “nudge” consumers towards healthier choices (Kawa et al., 2022; van der Laan & Orcholska, 2022).

Food packaging is an environmental cue which can influence food intake (Chu et al., 2021). While the primary function of packaging is to protect its contents, it also offers a means to communicate with consumers (Farmer, 2013; Lo et al., 2017). Consumers associate packaging with the contents (Ahmed et al., 2005), and this becomes integral to the product. Packaging serves as a marketing communication tool to shape consumers' product perceptions (Gil‐Pérez et al., 2020; Schnurr, 2019; van Ooijen et al., 2017) and behaviour towards the product (Koenigstorfer et al., 2013; Ribeiro et al., 2018; Spanos et al., 2015). Perception of the package is formed by integrating physical and functional elements such as recyclable, high quality, safe and attractive (Venter et al., 2011). An attractive package, with familiar branding, can encourage children to eat more healthful foods (Cravener et al., 2015; Keller et al., 2012). However, cartoon characters and brand mascots are often used to promote HED foods (García et al., 2019). Thus, there have been calls for on‐pack cartoon characters to be banned to avoid excessive consumption and this has been done in Chile (Campbell, 2022). This illustrates the power of packaging to affect food choice and intake. Clearly, packaging does more than protect product contents; it is an integral part of the product and can be used to communicate to consumers.

Studies manipulating packaging features of HED foods have used different labelling systems (Cecchini & Warin, 2016; Chantal et al., 2017; Gibson‐Moore & Spiro, 2021), on‐pack serving suggestions and small sizes to nudge reduced intake (Chu et al., 2021). For example, Madzharov and Block (2010) demonstrated that fewer product units displayed on packaging reduced intake intention. Similarly, for low‐energy dense (LED), nutrient‐dense foods, Cravener et al. (2015) applied cartoon characters and stickers to containers to promote vegetable intake in children. Children were assigned to a treatment or control group. Children in both groups received vegetable and granola bars in generic packaging during the first week, then the treatment group had 2 weeks of vegetable snacks presented with a favourite cartoon character on the container and a sticker inside the container. The control group continued to receive the generic packs. During week 2, children in the treatment group ate more vegetables than children in the control group. At the same time, the treatment group also chose the HED granola bar less than the control group during weeks 2 and 3. Simply providing a “fun” element to the packaging and a reward in the form of a sticker increased vegetable intake in children. Pires and Agante (2011) transferred elements typically used to promote HED snack foods to packaged apple slices including strong primary colours, “fun” fonts and a cartoon character to appeal to children. Significantly higher purchase intention was shown for the “fun packaging” compared to the same food presented in regular packaging. However, applying brand mascots and cartoon characters to promote HED foods such as cookies, confectionery and cereal is significantly more effective in directing children's food preference, choice and intake than these same techniques applied to fruits and vegetables (Kraak & Story, 2015).

Experimental evidence has demonstrated that manipulating different elements of packaging has the potential to affect how much food is eaten. Thus, changes to on‐pack cues such as images on the front of the pack, showing a small portion or structural features such as small packs, as well as partitioning and resealability all contribute to reduce food intake (Chu et al., 2021; Tang et al., 2020, 2022). Taken together, packaging features can be used to limit intake of HED foods and the transfer of some successful marketing strategies to packaging such as cartoons, “fun fonts” and appealing colours can encourage intake of LED foods.

The visual environment around product packaging is a strong driver of consumer behaviour, if the features grab attention and are noticed. At rapid decision speeds, visual saliency influences choices more than preferences do, independent of consumer goals (Milosavljevic et al., 2012). Many packaging elements, including visual attributes and provision of information, have been investigated to identify features which can attract consumers to the product (Waheed et al., 2018; Zekiri & Hasani, 2015). Clement et al. (2013) found that structural features (e.g., shape) and contrast dominate the initial phase of visual searching. Brand or logo‐related and graphic elements of packaging are salient (Cholewa‐Wójcik & Kawecka, 2015), whereas nutrition‐related elements are visually inconspicuous (Orquin et al., 2020). These findings demonstrate some barriers to consumer attention and yet a potential opportunity to guide visual attention and therefore food purchase decisions, for example, drawing attention to nutrition content or claims using structural features and contrast.

Visual attention to packaging features, however, does not necessarily align with preference. For example, in an eye‐tracking study, Husić‐Mehmedović et al. (2017) found that packages which attracted most attention (greatest number of fixations on the product measured by the eye‐tracker) were not the most liked. Indeed, memory for packages at the end of the experiment did not match the number of fixations. Participants were more likely to recall certain brands rather than those they looked at most (Husić‐Mehmedović et al., 2017). This suggests that the relationship between attention, liking and recall of packaging is more complex than a simple attentional capture process.

Therefore, while the laboratory offers an ideal context for controlled studies of packaging features and their effect on intake (Chu et al., 2021) and for measuring visual attention, such as eye‐tracking (Husić‐Mehmedović et al., 2017) it is limited for understanding consumer motivation and real‐world packaging purchase and use. On the other hand, outside the laboratory, where consumers make everyday decisions about food, there is debate around whether decisions are deliberative and open to explicit account or intuitive and more implicit in nature (Newell & Shanks, 2014). For the current study, a qualitative and exploratory method, photo‐elicitation, was undertaken to promote a deep level discussion around products already purchased by participants to elicit insights and explanations (Blinn & Harrist, 1991; Prosser & Schwartz, 1998), with the caveat that this method relies on recall.

The aim of the study was to investigate packaging features that may promote healthy eating. To achieve this aim, participants were invited to describe the elements of packaging of food items they had already purchased, structured around the following research objectives: to explore packaging design features that were salient to consumers; to identify particular features of packaging design which consumers associated with portion control; and to elicit suggested improvements to packaging for a co‐produced framework for designers to promote healthy eating.

## METHODS AND MATERIALS

### Research design

Photo‐elicitation provides a rich source of data, especially around the visual dimension (Harper, 2002), and can capture everyday perspectives of participants (Richard & Lahman, 2015). There are two variants of photo‐elicitation (Van Auken et al., 2010). One is researcher‐driven, where preselected photographs are presented to initiate discussion, and the other is participant‐driven photo‐elicitation (PDPE). Here participants take photographs to help address preselected research questions. PDPE encourages participants to have control in the interview by interpreting or discussing the photos they took and can help to prompt memory (Loeffler, 2004; Van Auken et al., 2010). The technique is widely applied in Anthropology, Sociology, Psychology (Dowdall & Golden, 1989; Green et al., 2021; Harper, 2002) and Design where it is called “culture probes” (Gaver et al., 2004; Mattelmäki, 2005). In recognition of the potential of photo‐elicitation to foster exploration and personal connection with the images (Hatten et al., 2013), this study employed PDPE to address the following research questions: (1) what features of packaging design are noticed by consumers, (2) which packaging design features are important for portion control, and (3) how can design be improved to contribute to healthy eating. The protocol was approved by the School of Design Ethics Committee (LTDESN‐139).

### Participants

Recruitment was conducted via social networks, word of mouth and the University Participant Pool. Participants were informed of the aim and that the focus was on food/drink packaging. Participants had to be aged over 18 years and had to give informed consent after reading the information sheet with details of secure data storage, anonymisation of data and the right to withdraw. Based on previous studies of photo‐elicitation with young people on food‐related issues, we aimed to achieve a sample size of at least 30 (Green et al., 2021). In total, 32 participants agreed to take participate, but seven participants withdrew from the study: some mentioned the effects of the pandemic and others gave no particular reason (this was an option on the consent form).

The final sample consisted of 17 females and 8 males, who completed all three tasks in the study. The participants were aged between 20 and 32 years of age ranging in nationality (16 Chinese; 7 British; 2 others). Most of the participants (23/25) were full‐time University students (23/25) and had a bachelor's or higher degree. According to their self‐reported heights and weights, most participants were in the healthy weight range (64%: *n* = 16) and for 28% of participants (*n* = 7) body mass index was in the overweight range. The two remaining participants were classed as underweight. Two‐thirds of participants reported that they were not on a diet to lose weight (see Table 1).

**TABLE 1:** Descriptive statistics characterising the participants.

Participants	Mean ± SD (*n* = 25)	Range
Female: Male ratio	17:8 (68%:32%)	–
Not dieting: dieting ratio	15:10 (60%:40%)	–
Age (years)	25 ± 3	20–32
Weight (kg)	65 ± 13	48–90
Height (cm)	167 ± 9	152–189
BMI (kg/m^2^)	23 ± 3	18–29

### Procedures

Following initial interest in the study, a link was sent by email with information and the consent procedure. After consent had been granted online and participants had provided demographic information, each participant was instructed to complete three tasks. The first two tasks involved PDPE, in which participants were asked to take 10 photographs in total. Five photographs were of packaging with salient (“eye‐catching”) features drawn from items consumed at breakfast, another meal (lunch or dinner), snacks (one salty, one sweet) and a beverage. Then, a further five images were requested to represent packaging which had helped the participant attend to portion or serving size across the same meal/snack contexts. If participants were not able to provide this, they were asked instead to use an image to illustrate where portion size was confusing.

**FIGURE 1:**
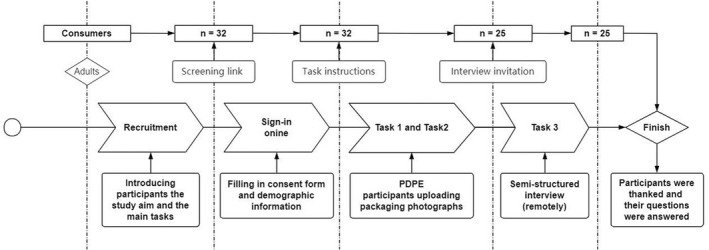
Diagrammatic representation of the research timeline and procedure

Each participant was provided with a secure online link to upload his or her images. Once the photographs had been uploaded, the third task was arranged, consisting of an online semi‐structured interview to discuss these images via Zoom or Microsoft Teams. The interview began with an invitation to describe each photograph and the reasons for selecting this. Then, a discussion of each image followed the sequence of meal context (breakfast, meal, snacks and beverage). Finally, open‐ended questions were asked to gain more information and insights about how packaging could help with portion control from participants, and each had the opportunity to add anything further they wished to say about how packaging can be improved. All interviews were audio‐recorded with permission (see timeline in Figure 1).

### Data analysis

Interviews were transcribed so that the data could be analysed, and transcriptions were checked for clarity and comprehension. Each participant was given an ID based on his or her demographic information. All names and identifying information were removed from recordings. A qualitative, thematic analysis (Braun & Clarke, 2014) was conducted by the first author to identify common or contradictory issues across interviews which were then discussed by all authors. Any consensus or dissent across interviews and any implications for packaging design were interpreted in detail by all authors. All data were iteratively coded and analysed using NVivo (version 12, QSR International Software, Melbourne, Australia). Discussions were conducted among authors to consolidate the main themes identified and to consider how to interpret the data.

## RESULTS

Participant ID is presented alongside quotations with a letter and digit combination then sex (male [M] or female [F]) and dieting status (dieter [D] or non‐dieter [ND]). This was done to provide a context for the quote.

### Task 1 ‐ salient features of packaging

Participants described the features of product packaging according to what was attention grabbing. The main themes raised were building trust, stimulating appetite and connecting to self‐identity.

#### Trust building

Attention was drawn to features that elicited feelings of trust. For some, packaging represented a means of building trust between them and the food products (see Table 2). For example, transparent panels provided reassurance about contents. Being able to see the “real” content rather than an idealised rendering of the product was helpful and authentic. In addition, some verbal descriptions (e.g., “pure,” “premium”) drew attention by promoting ideas of “quality” of the contents. Images representing flavours or ingredients were appealing to some participants, in part, as “guarantee” of the content and as convenient communication without words. Trust was also identified by the use of text conveying content indicating quality and inferring freshness or provenance for ingredients (e.g., showing the principal flavour of fresh fruit for jelly; image 2d).

**TABLE 2:** Photographs and elicited commentary: building trust

**Transparent element**		
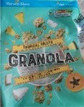 Image 2a	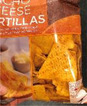 Image 2b	I suppose there is a see through in the middle, so you can see what is actually in it. And you can compare it with the picture. In the middle, (it) makes you focus on the real product. (CP_17_M_ND) (image 2a) I can see both the food image on pack and the real food inside the pack through the transparent window. And they are similar which wins more trust from me. (CP_07_M_ND) (image 2b)
**Realism of the food image**		
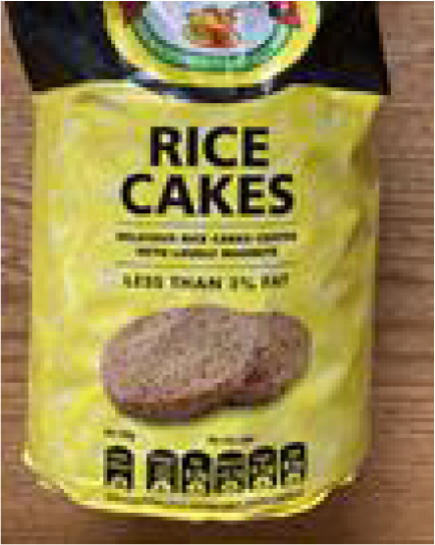 Image 2c	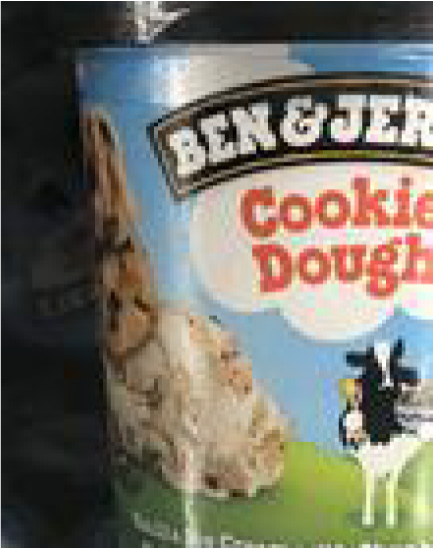 Image 2d	The image of rice cakes is close to the real product which is attractive, it is similar like the transparent packaging that I can see what the products looks like. (CF_24_F_ND) (Image 2c) Also, there is an image of the real ice cream which makes me feel it is quite delicious. (CF_25_F_ND) (Image 2d)
**Quality claims**		
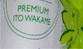 Image 2e	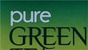 Image 2f	There are lots of seaweed I could pick, but I think it also says “PREMIUM” on the top pf the packaging as well, so I will say it is in good quality. (CP_13_F_ND) (Image 2e) It seems like there are a lot of “pure” elements on the packaging. (CP_14_M_D) (Image 2f)
**The image of ingredients or flavour**		
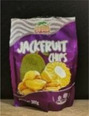 Image 2g	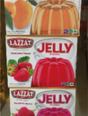 Image 2h	The ingredient of the food has been shown makes me know quickly about it and trust it. (CP_05_F_ND) (Image 2g) It shows the original ingredients, the comparison between the original food and the product inside makes it more attractive, I think. The picture of jelly is quite real. (CP_15_M_D) (Image 2h)

#### Appetising

Packages could stimulate appetite with prominent visual features including bright and contrasting colours, food rendering and enticements to eat (see Table 3). Participants considered bright colours (e.g., red, yellow, green, orange) as eye‐catching and combinations of contrasting (bright and dark) colours as especially attractive. Packaging colour promoted the perception of high‐quality and good taste. Participants were drawn to colours of packaging which matched the flavour of the food/drink contents (congruency). In addition to the use of colour, having a visual representation of the cooked meal or food was noted as attractive to participants. The rendering was enticing and appetising (image 3c), with a finished product after cooking or preparation described as arresting and “delicious” (image 3d).

**TABLE 3:** Photographs and elicited commentary: appetising features.

**Food renderings**		
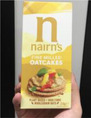 Image 3a	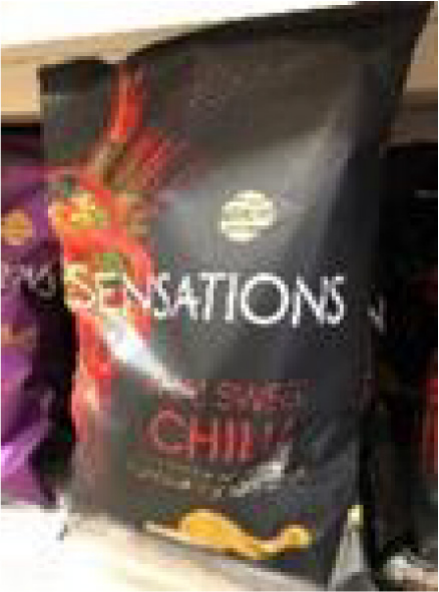 Image 3b	The yellow colour used in the whole package makes me feel it is very refreshing. This colour also increased my appetite. (CP_15_M_D) (image 3a) This packaging could increase my appetite. The combination of the black and red colour makes it high in quality, quite different from other crisps, just a feeling. (CP_08_F_ND) (image 3b)
**Bright or contrasting colour**		
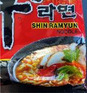 Image 3c	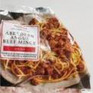 Image 3d	It showed what the food would be like after the processing which increased my appetite. (CP_04_F_D) (Image 3c) The image of the food after it has been processed. I could not stop imagining how delicious it would be. (CP_05_F_ND) (Image 3d)

#### Self‐identity

Some packaging elements connected to consumers reflecting values and identity, for example, pursuing health goals or showing interests in their own or other cultures (see Table 4). Making nutrition claims such as having less sugar or fat or more fibre content (image 4a) were identified as important to participants with health motives. Participants on a diet attended to descriptions aligned with their goals to achieve a healthy diet. Also, cultural identification from text, national imagery or symbolism was noted. Participants were attracted not only to packaging with elements from their own culture but reported that features of other cultures (image 4d) were also eye‐catching and appealing.

**TABLE 4:** Photographs and elicited commentary: packaging, values and self‐identity.

**Nutrition and health claims**		
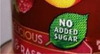 Image 4a	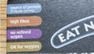 Image 4b	There is no added sugar in it. I do look for that, so, this is “no added sugar.” (CP_13_F_ND) (image 4a) The different coloured label on the side, it kind of tells me it is for veggies, high in fibre and there is no refined sugar. So, I am quite interested it because I am trying to eat as healthy as I can. (CF_24_F_ND) (image 4b)
**Culture interests**		
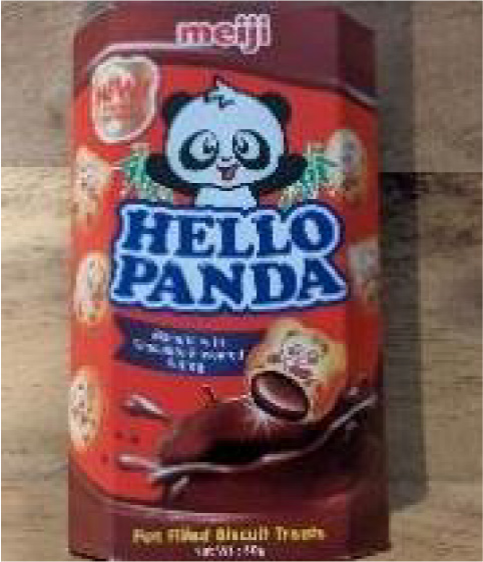 Image 4c	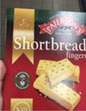 Image 4d	There is a nice panda logo, a kind of Chinese style. (CP_14_M_D) (Image 4c) The packaging gives a kind of Scottish feeling, the red colour and the green colour that like reminds me (of) the Scottish kilt. (CP_19_F_ND) (Image 4d)

Overall, these results indicate the prominence of colour, textual or pictorial descriptions, transparency, rendering and cultural associations. Noticeably, serving size was not mentioned by any of the participants during this PDPE task, suggesting that this was not salient to the participants.

### Task 2a ‐ focusing on portion/serving size

In Task 2a, participants were asked to take photographs, which had helped them attend to the portion or serving size (and if they could not do this to showcase features which hindered portion control). Themes identified ranged from structural reminders to healthy prompts and raising portion awareness.

#### Structural reminder

Structural features of packaging served as helpful reminders to limit intake to a single portion/serving size (see Table 5). For example, packages containing multiple small packs were described as preventing excess intake (image 5a) and pre‐packaged servings kept the product fresh, and limited intake (image 5b). In addition, individual servings in small containers were considered a physical aid to portion control (image 5c). Participants reported that they usually consumed less when purchasing small, individual packs, recognising these as convenient and less wasteful.

**TABLE 5:** Photographs and elicited commentary: portion reminders.

**Pre‐packaged portions within large pack**		
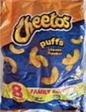 Image 5a	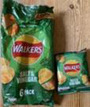 Image 5b	This product includes eight packs. I understand that one pack (is) just for one serving. Actually, they did not say anything about serving portion, but I can understand that one pack is just for one time as a snack. (CP_11_F_D) (image 5a) The individual package of the crisps will at least guarantee that I can eat a small portion rather than eating too much at once. (CP_19_F_ND) (image 5b)
**Individual servings**		
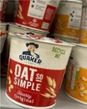 Image 5c	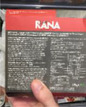 Image 5d	These are smaller pots, I can use these if I am going to work, I can take this small pot with me. One pot one portion. (CP_23_M_ND) (Image 5c) As you can see, it is a handful package. It is quite small. I can hold it with one hand. So, I guess it is perfect for one portion. (CP_15_M_D) (Image 5d)

#### Healthy prompts

Packaging promoting contents as low in energy and high in healthiness influenced purchase and intake decisions (see Table 6). On‐pack energy (kcal) content with clear numbering was recognised as an important and salient cue to promote portion control (images 6a and 6b). In addition, front of pack or back of pack nutrition and energy content were mentioned as useful portion guidance. Traffic lights were highlighted as important, with red serving as a warning for high content of one or more macronutrients (fats, sugars) and green or amber for low or moderate levels. Healthy prompts, which were simple, salient and clear, appeared to be valued.

**TABLE 6:** Photographs and elicited commentary: representing packaging features that promote low‐energy content or healthiness of foods.

**Independent energy content**		
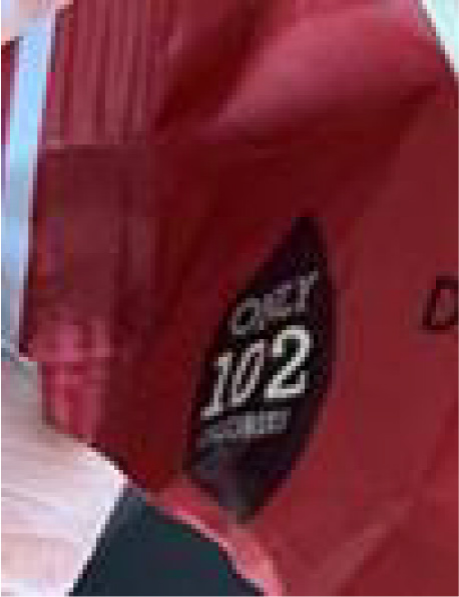 Image 6a	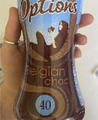 Image 6b	It is more appetising as a healthy snack. The fact is that on the packaging it says it is 102 calories, very clearly. (CP_20_F_D) (image 6a) If you were cautious about the portion size, it has the “40” at the bottom. It means it is 40 calories, if you are cautious, it will be there for you to notice that it is like a low calorie. So, I think it is a good option for hot chocolate. (CP_21_F_ND) (image 6b)
**Traffic lights**		
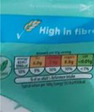 Image 6c	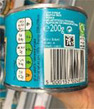 Image 6d	On the top of the packaging, you got different colours, red, amber or green on the amount of the nutrition information. When you see those things, you will think, ok, maybe the portion size should be smaller. So, it is quite helpful. Especially, when it is something red on it. (CP_17_M_ND) (Image 6c) It clearly shows the nutrient contents of the whole can. To me, it is a kind of standard portion for one sitting. There is no red label which means it is quite healthy. (CF_25_F_ND) (Image 6d)

#### Portion awareness

Transparency and on‐pack suggested serving size or unit number were considered helpful (see Table 7). The transparent panel showed how much food remained in the pack, assisting with planned intake. The on‐pack unit number was also recognised as an important cue for portion size, even when these units were not pre‐packaged.

**TABLE 7:** Photographs and elicited commentary: representing packaging features that raise portion awareness.

**Transparency**		
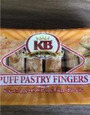 Image 7a	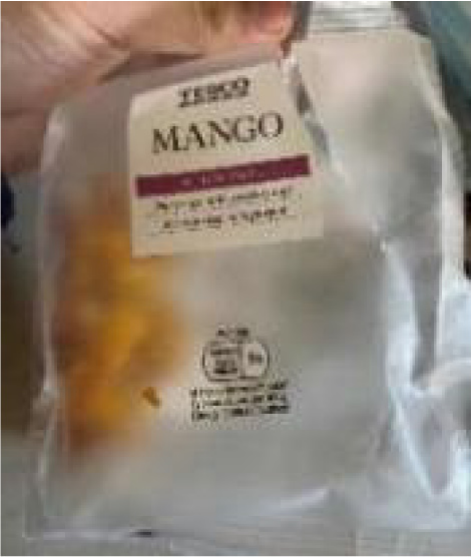 Image 7b	The packaging is partially transparent. I can see the puff biscuits inside and I think there are quite a lot and can be consumed multiple times. (CP_02_F_ND) (image 7a) It is transparent, you can easily observe how much you eat and how much is left. (CP_03_M_D) (image 7b)
**On‐pack unit content**		
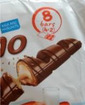 Image 7c	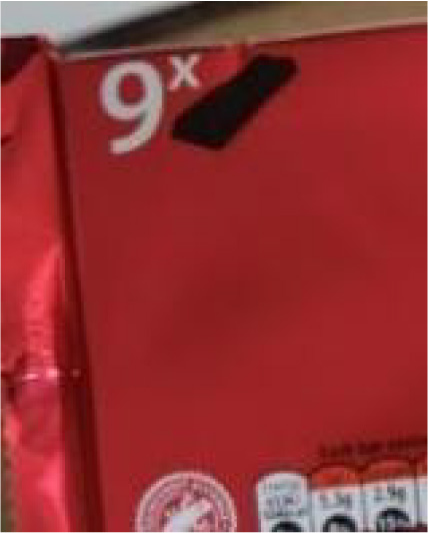 Image 7d	It includes eight bars. It means one bar is equal to one portion. I notice the number “8” on the packaging, so I know the meaning. (CP_11_F_D) (Image 7c) It tells me there are nine bars inside the packaging. So, I know I may eat just one bar each time. (CP_10_F_ND) (Image 7d)

### Task 2b ‐ unclear and confusing portion

Misleading or confusing information was noted when portion guidance was omitted, or there were no obvious means to portion out products (see Table 8). Portion guidance was unavailable on some products, for example amorphous foods such as jam or where many units were contained in a single, large pack such as candies or nuts. Without specific serving sizes, participants reported confusion about appropriate portions for a single sitting and in some cases believed that this omission encouraged overeating and caused guilt. Furthermore, some food packaging did provide nutrient information and serving recommendations, but this was hard to read, understand and apply. For example, text was too small or not obvious to find, or where it was given, it was hard to equate from weights to domestic measures (e.g., what does 30 g of this food look like?) This prevented participants from following the portion instructions.

**TABLE 8:** Photographs and elicited commentary: packaging features that arouse confusion about the portion.

**Unavailable portion guidance**		
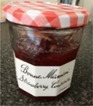 Image 8a	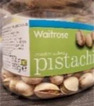 Image 8b	It is not controllable because it did not say anything about the serving. How many spoons of this jam will be enough for one person? It did not say anything. That's why sometimes, I feel I eat too much and feel guilty. (CP_11_F_D) (image 8a) The roasted pistachio tastes really good, once you start, it is hard to stop. … I am not sure if there is any suggested amount for each time. Just imagine that you are watching TV, having fun with your friends, it is really easy to overeat. (CP_09_F_D) (image 8b)
**Inconvenient to portion out**		
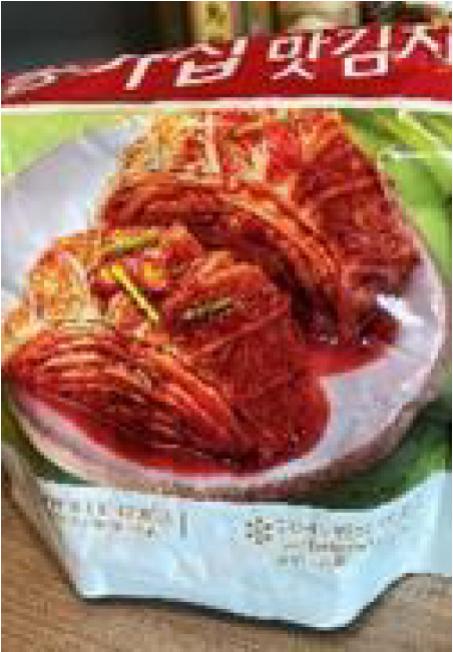 Image 8c	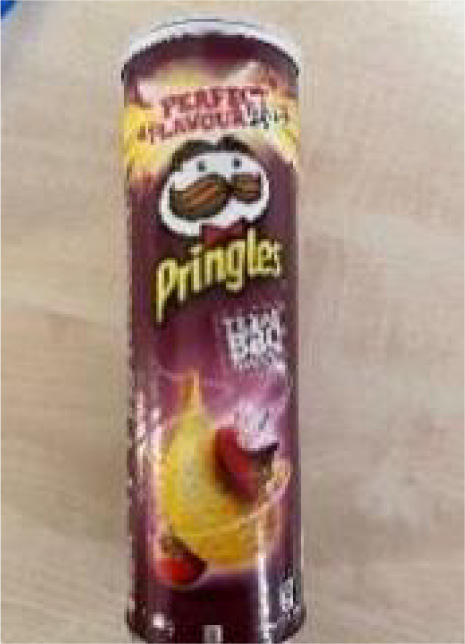 Image 8d	The nutrition and serving size information is on the back, not very obvious. The on‐pack image also has nothing to do with the portion size or something. So, I think its portion size is quite confusing. (CF_18_M_ND) (Image 8c) Although one serving is about 30 g which is written near the ingredient table, the number still needs to be further calculated, which is difficult to know what is the portion it refers to. If I consumed a handful of chips, I cannot intuitively link the portion to the gram. (CP_01_F_D) (Image 8d)

### Task 3 ‐ improvements to packaging design

Ideas to promote portion control and healthy eating were offered by participants (see Table 9). A consensus was noted for clarity of messaging around healthiness of the product, providing obvious and easy to follow portion size information (e.g., using illustrations or everyday objects such as hands), providing single serving and multiple sub‐packs, and using equivalence in visualising content (e.g., presenting protein content as equivalent number of eggs).

**TABLE 9:** Participant suggestions to improve packaging design for portion control.

Suggestions	Quotations
Highlighting the healthiness of the product	I hope the packaging can better reflect the healthiness of food, highlight the low calories if it is. I think it will be so helpful to the people who care about healthy eating. (CP_15_M_D) I would say maybe the food can tell you it is really high in fat or high in salt in big, bold letters. Because I think currently it is just the tables on the bottom, if it is really high in fat, it should be in big bold letters. Sometimes, the food may be considered as healthy food, but they are actually not… so it is necessary to let people aware and understand it. (CP_22_F_ND)
Presenting obvious and clear portion related information	I think the portion related recommendation should be highlighted. Make them more obvious, like on the front rather than on the back. Sometimes I need to find really carefully some kind of recommendations which could be improved. (CP_10_F_ND) Some unpartitioned products may give the nutrition information for each serving, however, I used to be misled that it is the calorie of the whole pack. I would think it is so healthy because of the small numbers, then I happily ate the whole pack without any concern. But the truth is I had too much energy intake. I hope such things could be avoided somehow. (CF_25_F_ND)
Providing single serving	A smaller portion, a handful size maybe, will let people have a sense that it is for one serving. (CP_15_M_D) It is hard to find a smaller portioned product that is suitable for one person. Once it is opened, you can never keep the taste the same as before…you will just eat the whole big pack without noticing, you cannot imagine how many calories you consumed. (CP_12_F_D)
Offering multiple sub‐packs	I do hope food could be divided into small packs or for a single serving. Like the biscuit, it will be helpful if fewer pieces are in a small pack. (CP_04_F_D) The Kinder chocolate, when we open this product, we just face different separate packages. I think it is a good strategy to control the portion. Also, it will be beneficial for keeping the product fresh and its flavour. (CP_11_F_D)
Understandable ways to present the portion suggestion	Find more ways to show the recommended portion or remind people about the intake, like providing other images as references. (CP_05_F_ND) Quantify or visualise the ingredients to something people are familiar with. For example, if the food is high in protein, I would like to know it is equal to how many eggs? Also, the calories in numbers. It is hard to understand. I prefer that the packaging could tell me the calorie of this food equals how many bowls of the rice. I think it is a more direct way to explain such information. (CP_08_F_ND)

## DISCUSSION

**FIGURE 2:**
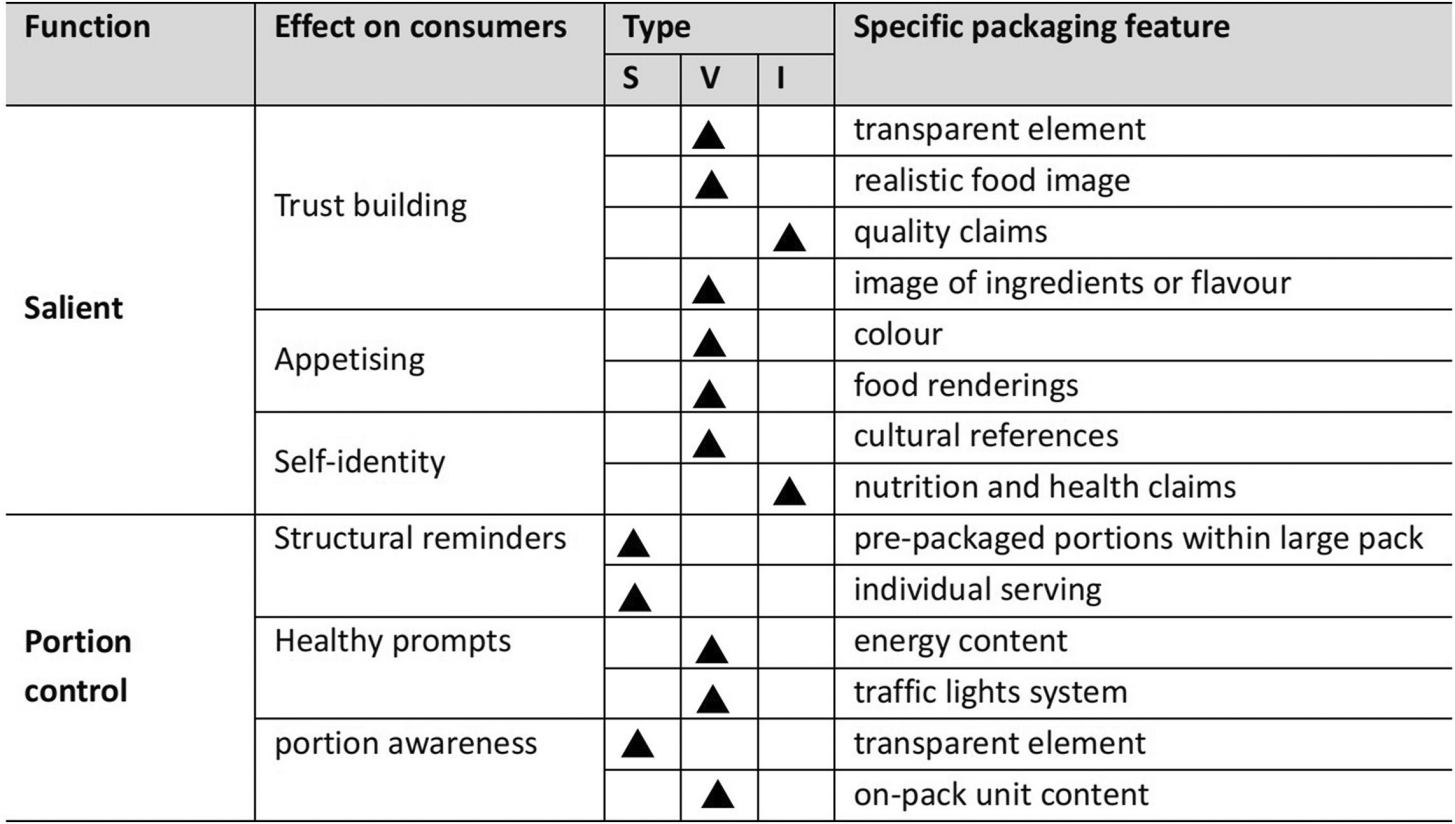
Salience and portion control themes identified by participants grouped by structural (S), visual (V) and informational (I) features. *S = structural (The physical characteristics of the packaging, mainly in terms of space, material and shape); V = visual (Packaging elements that are more visually prominent and have a stronger visual impact compared to other attributes); I = informational (Packaging with elements that convey a specific message and a certain implication) (Packaging features were only grouped into the type that were more prominent in the participant responses.)

A variety of packaging features were identified as salient around building trust, making foods appetising, or linking to goals and identities of participants. Furthermore, participants identified structural reminders, health prompts and portion awareness as packaging features that could enhance portion control. Participants raised issues around lack of clarity on packaging about serving sizes and nutritional information and they gave several suggestions to improve packaging. A summary of these from the participants has been provided in Figure 2.

These findings offer preliminary steps for building a framework to improve packaging design to support healthy eating by, for example, making portion control features more prominent; enhancing the appeal of healthy food; and capitalising on what consumers notice most to communicate portion sizes clearly. However, this framework must be considered with caution, since participants in the present study are not representative of the general population, given that they were on average young, with a healthy bodyweight and well educated. The participants appeared to be motivated by healthy eating goals according to the transcripts. Therefore, the findings must be considered in light of the highly selective nature of the sample obtained. In addition, while the participants identified ways that packaging encouraged trust‐building, for example, consumer research indicates a lack of trust by the public both in the portion sizes suggested on the pack (IGD, 2019) and in food labelling (Tonkin et al., 2016). Indeed, the use of images of fresh fruit, for instance, on foods that are processed, such as jelly, could be considered misleading, even if it is to convey a flavour.

### Enhancing structural and visual features that helps portion control

Previous research indicates that structural and visual features of packaging are more likely to facilitate portion control than information. These features can be applied to limit intake of HED food. For structural features, individual serving sizes aid portion control. Similarly, pre‐packaged portions within a larger pack provide a structural reminder for consumers to eat only what is contained in the pack or partition, the so‐called “segmentation effect” (Geier et al., 2006; Kerameas et al., 2015). People value convenience (Candel, 2001) and want choice to be effortless (Scholderer & Grunert, 2005). Thus, pre‐portioned units promote portion control (Rolls et al., 2004) since these are convenient and effortless, removing deliberation from the process (Coelho do Vale et al., 2008). Interestingly, small packages and partitions promote purchase intention in that consumers feel more in control and predict less food waste (Petit et al., 2020). Yet, this solution raises concerns about the environmental cost of additional packaging. Tang et al. (2022) have designed some examples of promoting sustainable, reusable and/or recyclable materials for small, pre‐portioned packs, which provide one way to avoid food waste and reduce the environmental impact of packaging materials.

Reminders of low energy or high nutrient content were considered helpful if presented in an attractive way. Simple numbers and traffic lights systems make healthiness obvious and visible (André et al., 2019; Carrillo et al., 2014). Participants made a particular note of the red traffic light serving as a reminder to limit intake (Lunardo et al., 2021). Although consumers may ignore nutritional information or misinterpret this due to lack of knowledge, time, or motivation (Andrews et al., 2021; Spiteri Cornish & Moraes, 2015), packaging features can be optimised to nudge healthy choices (Tang et al., 2020, 2022).

A single unit is generally considered as the appropriate and culturally designated norm (Herman et al., 2005). Unit number or unit size serves as an on‐pack heuristic, nudging consumers towards a single portion size or unit. Transparency, either as a panel or cut‐out shape, was identified both as salient and as a means to promote portion control. The transparent panel is used by consumers to monitor intake and amount remaining. However, transparency may have opposing effects (Deng & Srinivasan, 2013), since seeing the product encourages intake (salience effect) and prompts intake surveillance (monitoring effect).

### Utilising salient features for healthy eating and downsizing

Applying visually attractive packaging features (e.g., colourful, alluring) usually reserved for HED items such as branding (Keller et al., 2012) to LED foods promotes healthy eating. Developing appealing and eye‐catching packaging features promote an immediate, positive first impression of the product (Silayoi & Speece, 2007). However, different elements on packaging receive various levels of attention from consumers. Previous studies (Cholewa‐Wójcik & Kawecka, 2015; Clement et al., 2013) have reported consumer brand, logo and graphic elements as visually salient for consumers. In the present study, consumer attention to packaging features included three further themes namely trust building, appetising and self‐identity.

Trust is important for consumers. Apart from the brand, and the label (Lassoued & Hobbs, 2015; Rupprecht et al., 2020), convincing descriptions and visual elements, such as a realistic rendering of the product or of key ingredients establish trust. Credibility and reassurance could be built from realistic visual presentations of what consumers would expect from the product, since consumers are more likely to believe what they see (e.g., through a transparent panel or rendered image) than what they read (Hoegg, 2015). As highlighted, trust plays a key role in food choice (Coveney, 2008). However, participants differ in how much they trust information displayed on the packaging, either because trust may have been affected by previous food scares (e.g., Wang et al., 2020) or as a function of health motivation (Siegrist et al., 2015). Also, trust may be affected by the extent to which some marketing techniques are perceived as misleading (García et al., 2019). Thus, any packaging design that enhances consumer trust in a product could be applied to nudge consumers towards healthy eating, but this will be mediated by individual and cultural differences and will depend on the veracity of visual representations or nutritional claims made on the packaging.

Packaging features that tempt appetite were identified as attention grabbing to consumers. Consistent with previous evidence, vivid colour (e.g., bright and contrasting colours) is attractive to consumers (Lee et al., 2013; Spence & Velasco, 2018). Therefore, if colours are congruent with the product, this could increase anticipated eating enjoyment and encourage desire to eat. Enticing images, like real food rendering, also serve to prime appetite. Experimental studies demonstrate that stimulating appetite contributes to food intake (Drapeau et al., 2005; Spanos et al., 2015; Zhou et al., 2021). This then presents a tension between the desire to achieve a healthy diet and the temptation to consume HED foods. On the one hand, stimulating appetite might be an effective marketing strategy that engages consumers' attention to the product and encourages consumption of nutrient‐dense foods. On the other hand, for consumers who wish to limit food intake for themselves or their children, the same strategy applied to HED foods makes them difficult to resist (Mehta et al., 2012).

Salient features of packaging also include those features that appeal to health goals or to cultural identities. People paid more attention to on‐pack health claims aligned to overall health‐related goals (Jung & Bice, 2019; McCarthy et al., 2017). For example, low‐energy content claims including low sugar and/or fat content are also salient to consumers with a desire to lose weight or to eat a healthy diet (Andrews et al., 2014). In addition, cultural connotations presented on packaging, like language, images, colours, were identified as salient as attentional bias relates to features which are personally relevant and important to them (Shen et al., 2015). This could be due to associations with home or a fondness for certain countries, formed through personal experience and preference (Bernard & Zarrouk‐Karoui, 2014). These suggest that the prominence of health‐related and personally relevant elements on packaging can encourage consumers to purchase and eat via personally relevant goals (Shafiq et al., 2011), including presenting images of athletes to denote healthy lifestyles (Schifferstein et al., 2022) and could be used on LED nutrient‐dense foods to encourage intake.

### Addressing the portion related information

Consumers noticed that serving size was unavailable for many products as revealed by Rippin et al. (2019), and others noted some products are inconvenient to portion out, for example, the serving size is given but poorly operationalised. Consumers want to be able to see information at a glance and apply with ease the portion prompts or serving recommendation on the packaging. To improve packaging design, participants also identified ways to promote more effective communication of recommended serving sizes.

Consumers wanted to have serving size information visually salient, specifically, for HED items given the competition among so many packaging attributes (Husić‐Mehmedović et al., 2017). Like the relative surface size, position (e.g., the distance to centre) and the contrast (e.g., the contrast with background colour) (Orquin et al., 2020; Peschel & Orquin, 2013), portion recommendations need to be presented so that they are prominent. Taken together, these suggested improvements in visual salience could be used in design, combining the reported attention‐grabbing elements with portion size aids to guide the consumer more effectively. Some nutrition terms are unfamiliar to consumers which makes communication challenging (Spiro & Wood, 2021). In the present study, creative domestic measures were preferred over weights in grams or energy content in kcal. Simple visual images using hands (e.g., Benelam & Stanner, 2019) could represent recommended portions since the image requires less cognitive effort to process than numbers and text (Smith et al., 2015).

### Design implications

Insights from participants could guide designers to improve food and beverage packaging to meet their expectations (Ampuero & Vila, 2006). Thus, three main strategies within an implication framework have been summarised below to guide further packaging design practices (see Table 10).

**TABLE 10:** Design implications to promote healthy eating

Design practical implications				Relevant features
Enhance portion reminders of HED food	Structural	→	Set structural reminder to prevent over consumption.	Pre‐partitioned packaging Single portion
	Visual		Raise the portion awareness to trigger effective self‐monitoring	On‐pack unit content Transparent panel
			Highlight the healthiness of the product with direct, clear and accurate signposting	Traffic light system Nutrition and health claims Independent energy content
Increase the packaging appeal of healthier food		→	Utilise the features that could contribute to trust building, make food appear appetising or cater to some healthy needs and identities to increase the attractiveness and the intake of nutrient‐dense or LED food.	Transparent element Realistic food image Quality or healthy claims Image of ingredients or flavour Vivid or high‐contrast colours Food renderings Cultural references
Better communicate portion‐related recommendations		→	Combine the portion size‐related information or image with visually salient elements to gain attention	
		→	Give prominence to the serving size recommendation to save time and effort to calculate this	On the front In the middle Large or bold font Vivid or high‐contrast colours
		→	Illustrate the portion information in grams or ml using specific measures which are easy to understand and follow	Equivalent physical activity effort Equivalent energy or nutritional content of foods Portions presented in easily identifiable measures, for example, hand size

Abbreviations: HED, high‐energy density; LED, low‐energy density.

There are increasing efforts to promote a shift towards more healthy diets (Bublitz & Peracchio, 2015). However, it is challenging to achieve health goals and simultaneously resist the temptation of HED foods (Fishbach & Zhang, 2008). Encouraging consumers to choose appropriate portion sizes is one way to reduce food waste and therefore is a positive contribution to the environment. However, pre‐packaged portioning may increase concerns about additional resources for package materials. Therefore, a balance is needed between promoting small portion sizes of HED foods using relevant, on‐pack, domestic portion cues and offering pre‐portioned packs using eco‐friendly materials (Tang et al., 2022).

Packaging offers a direct channel for capturing consumer attention and a means to communicate health and portion size and must be designed with environmental considerations in mind. Our co‐produced design strategies illustrate ways to build on existing social responsibility commitments to improve public health (Knai et al., 2018), offering consumers a convenient and sustainable way to support healthy eating. Packaging has a potential to nudge healthy eating, but this requires uptake by food companies and consumer awareness of public health campaigns to limit intake of non‐core HED foods (e.g., Public Health England Change4Life 100 kcal snack campaign; Day et al., 2022).

### Strengths and limitations

Photo‐elicitation is a method with some strengths, including providing a bridge between researchers and respondents based on lived experience. The photographs and their accompanying responses offer insights not normally accessed at a conscious level at the time of purchase decisions. However, asking participants to reflect on processes that may be more automatic than deliberative is problematic. Therefore, while participants were invited to respond to images they had taken during interviews, their response relied on memory and could be affected by recall bias as well as demand characteristics such as impression management. In addition, while the images were taken from packaging of typical meals and snacks, these represent a mere snapshot of an otherwise complex food system and substantial number of nutrition decisions. Other limitations of the study include the small sample size, youth and high education levels of participants compared to the general population of the UK, which restricts the generalisability of the findings. A potential strength of the sample was its inclusiveness, with a range of bodyweight status and more than half of the sample were originally from China, offering a unique perspective on packaging purchased in the UK. However, there are known differences between consumers from more collectivist (China) compared to more individualist (UK) societies and this may have influenced the outcomes of the study. For example, it has been reported that persuasive technologies to promote healthy eating behaviour have different targets for collectivists (e.g., perceived benefit) compared to individualist groups (e.g., perceived susceptibility, self‐efficacy) (Orji & Mandryk, 2014). Therefore, future research should include a larger, more diverse sample with participants drawn from different age groups, educational experiences and ethnic and cultural backgrounds.

Another limitation is that none of the participants raised portion size as a salient feature of packaging until asked to do so for the second task. It is possible though that they may have “saved” these comments for the second task rather than their omission reflecting lack of salience. A further limitation is that the suggested improvements to package design have yet to be tested systematically. Practical applications of these design improvements need to be conducted in a real‐world setting, with food companies who express an interest in the wider social responsibility agenda.

## CONCLUSIONS

Packaging attributes that participants found salient were around trust‐building, making contents appetising and linking to culture/values. Structural reminders, health prompts and convenient portion size images were noted as helpful for portion control. Adapting these packaging features for foods that are low in energy density and nutrient dense could increase their attractiveness and encourage intake. Improvements in how portion size is communicated visually coupled with physical features for downsizing will nudge consumers to support portion control. These findings from photo‐elicitation can be used to guide future improvements to packaging design. There is an opportunity for business to incorporate better packaging design for downsizing food intake to meet consumer needs, as well as to apply the portion control design framework to nudge healthy food intake.

## CONFLICT OF INTEREST

There are no conflicts of interest to declare.

## Data Availability

All data are available from the Open Science Framework (OSF) here ‐ https://osf.io/6zg9t/.
